# Supplementary material for: Filopodyan: An open-source pipeline for the analysis of filopodia
Source: J Cell Biol. 2017 Oct 2;216(10):3405–22. doi: 10.1083/jcb.201705113 (PMC5626553; doi:10.1083/jcb.201705113)
Supplement: Supplemental Materials (PDF) [file JCB_201705113_sm.pdf]

Urbančič et al., https://doi.org/10.1083/jcb.201705113

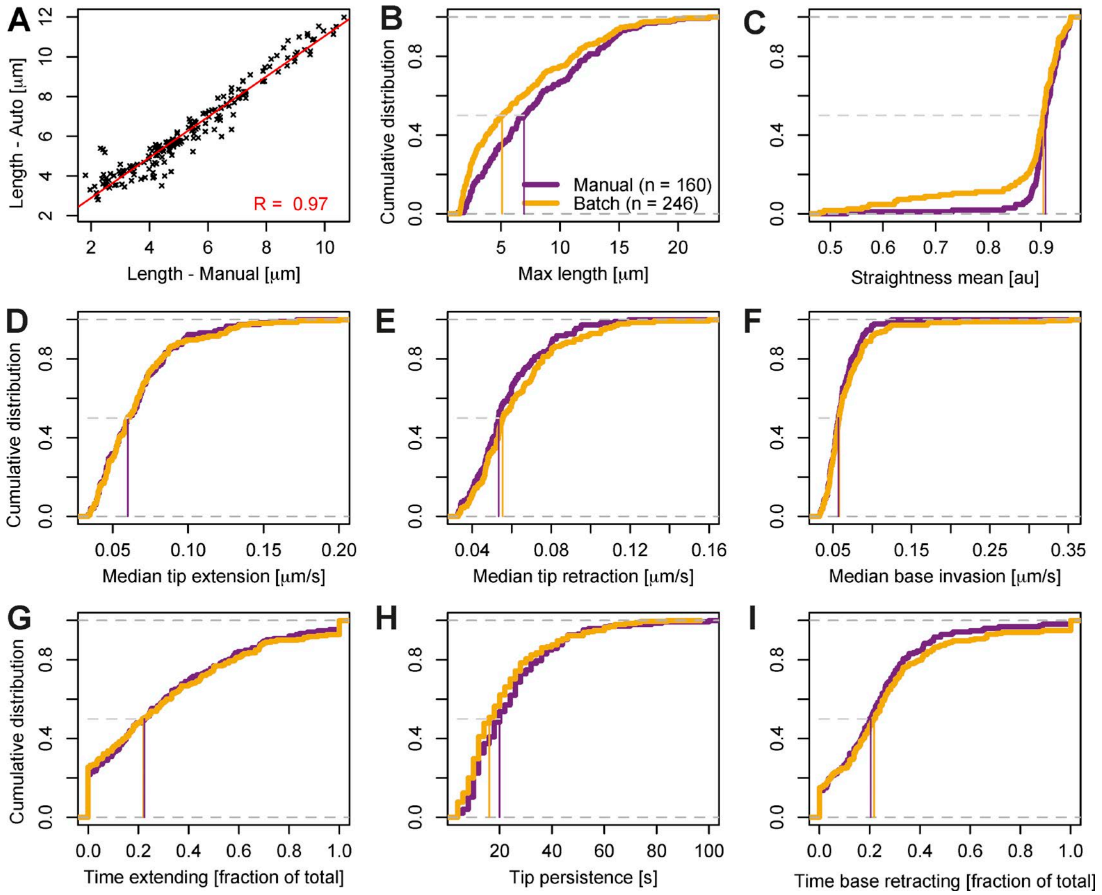

Figure S1. **Comparison of filopodia properties measured with and without manual editing of reconstruction.** (A) Correlation between 186 manual and automated measurements of filopodia lengths (6 filopodia, 31 time points each) from *Xenopus* RGC growth cone showing a strong good positive correlation between manual and automated measurements. (B–I) Measurements of filopodia properties from the descriptive dataset of 19 *Xenopus* RGC growth cones. Cumulative distribution curves are shown for the filopodia dataset following automated filtering only (yellow, CAD filter) and for automated filtering followed by extensive manual curation of the reconstructions (purple, manual filter). Median of each dataset is displayed as a vertical line intersecting the cumulative density curve at 0.5.

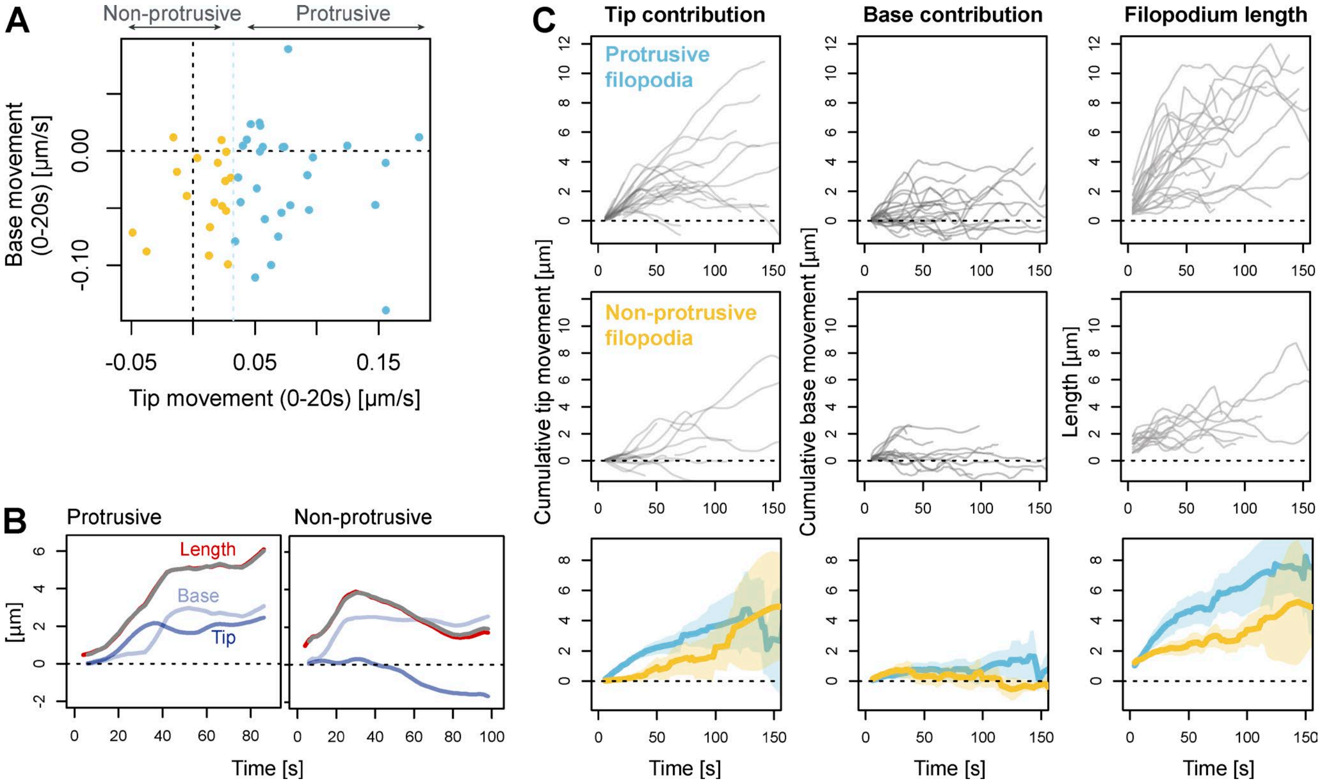

Figure S2. **Tip and base movements in filopodia arising from protrusive or retractive events.** (A) Scatterplot of median tip and base movement during the initial phase (first 20 s in existence) for newly forming filopodia (*n* = 45) in *Xenopus* RGC growth cones. Filopodia were divided into two categories depending on whether their median initial tip movement exceeded extension threshold (protrusive, blue, *n* = 28) or not (nonprotrusive, orange, *n* = 17). (B) Examples illustrating the dynamics of tip and base movement in filopodia in each category. Red line shows the total filopodium length (smoothed with five-step rolling mean). Blue line shows the contribution of tip movement to filopodium length (cumulative sum of smoothed DCTM measurements over the time series). Lighter blue line shows the contribution of base movement to filopodium (negative cumulative sum of smoothed DCBM measurements over the time series). Gray line shows the sum of cumulative tip movement and cumulative base movement, adjusted for initial length at the first available time point and showing good overlap with filopodium length. (C) Line traces for cumulative tip movement (left), cumulative base movement (middle), and total length (right) over time for all newly forming filopodia in the dataset, separated according to subgroup (top: protrusive; middle: nonprotrusive). Bottom row shows the mean and 95% confidence interval measurements for cumulative tip movement (left), cumulative base movement (middle), and total length (right) for each of the two subgroups of filopodia (blue, protrusive, *n* = 28; orange, nonprotrusive, *n* = 17).

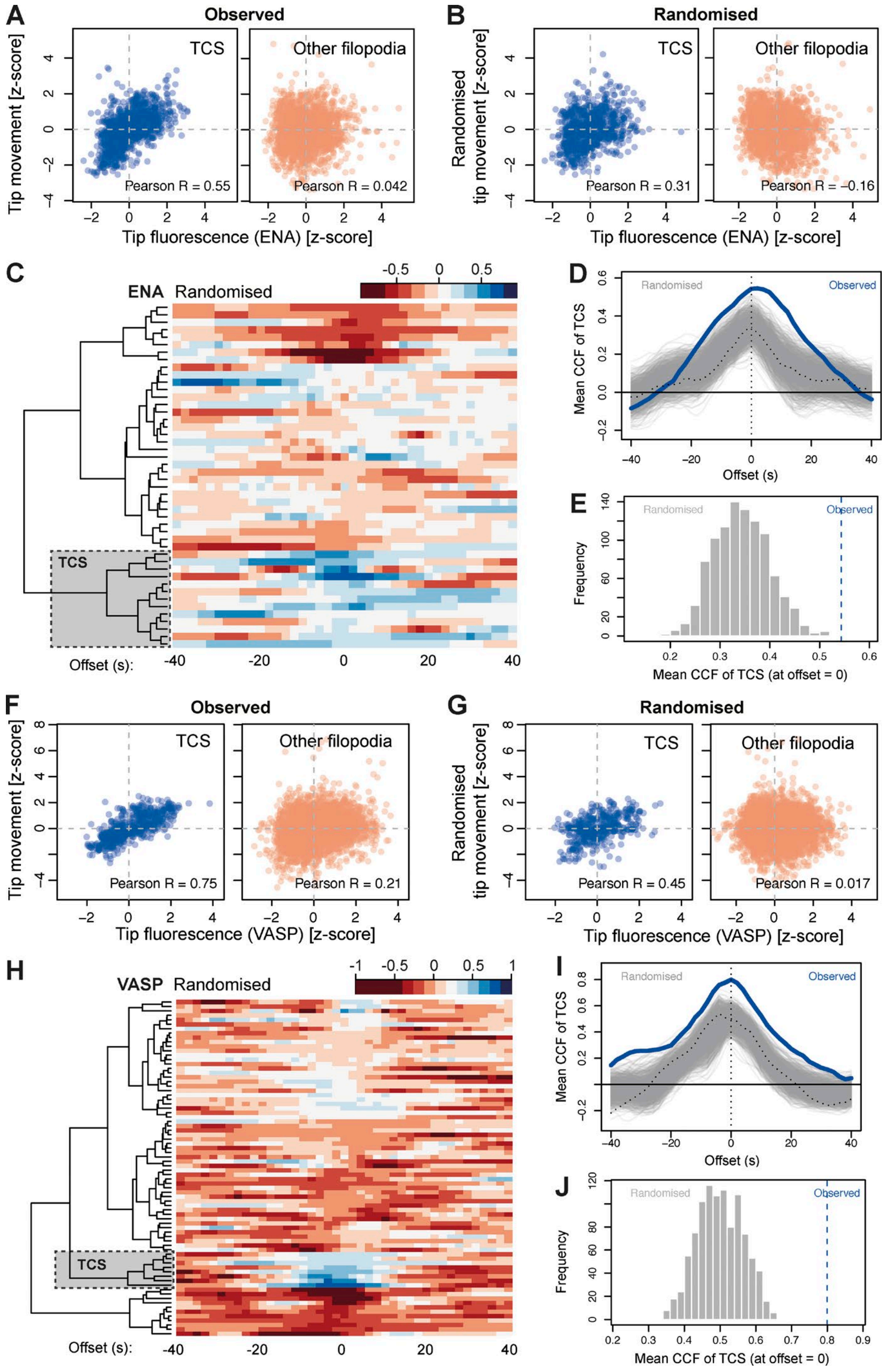

Figure S3. **Randomization control for the hierarchical clustering of cross-correlations between tip fluorescence and movement.** (A) Correlation between ENA fluorescence and tip movement for ENA-responding filopodia (blue; top-correlating subcluster in the CCF heatmap in Fig. 5 I) and for all other filopodia (red), normalized such that 0 = mean for each filopodium and 1 = SD for each filopodium. (B) Correlation between ENA tip fluorescence and randomized tip movement. After hierarchical clustering on the randomized dataset (in C) to identify the most positively correlating subcluster (top-correlating subcluster [TCS]), its correlation between fluorescence and movement is reduced by comparison to the original dataset. (C) Heatmap represents the level of cross correlation of tip movement and tip fluorescence for each filopodium after reshuffling the order of tip movement for each filopodium using block randomization to artificially decouple movement from fluorescence. Rows represent individual filopodia, columns indicate the offset (lag/lead) of the cross-correlation function. Gray boxed region indicates the top correlating subcluster (TCS) within the randomized dataset. Only one representative randomization out of 1,000 is shown. (D) Cross-correlation between (randomized) tip movement and ENA tip fluorescence in the top-correlated subcluster at different values of cross-correlation offset. Gray lines represent the top-correlated subcluster in individual randomized datasets (n = 1000), of which the representative example from C is indicated by dotted black line. Blue line (observed) shows the cross-correlation of the top-correlated subcluster in the original, nonrandomized dataset (as in Fig. 5). (E) Histogram showing the cross-correlation between ENA fluorescence and tip movement at zero offset for the top-correlating subcluster in all randomized datasets, with the cross-correlation of the original (observed) top-correlating subcluster indicated by the vertical blue line. After randomization, none out of 1000 positively correlating subclusters are cross-correlated as strongly as the original dataset ($P < 0.001$). (F–J) As in A–E, for VASP.

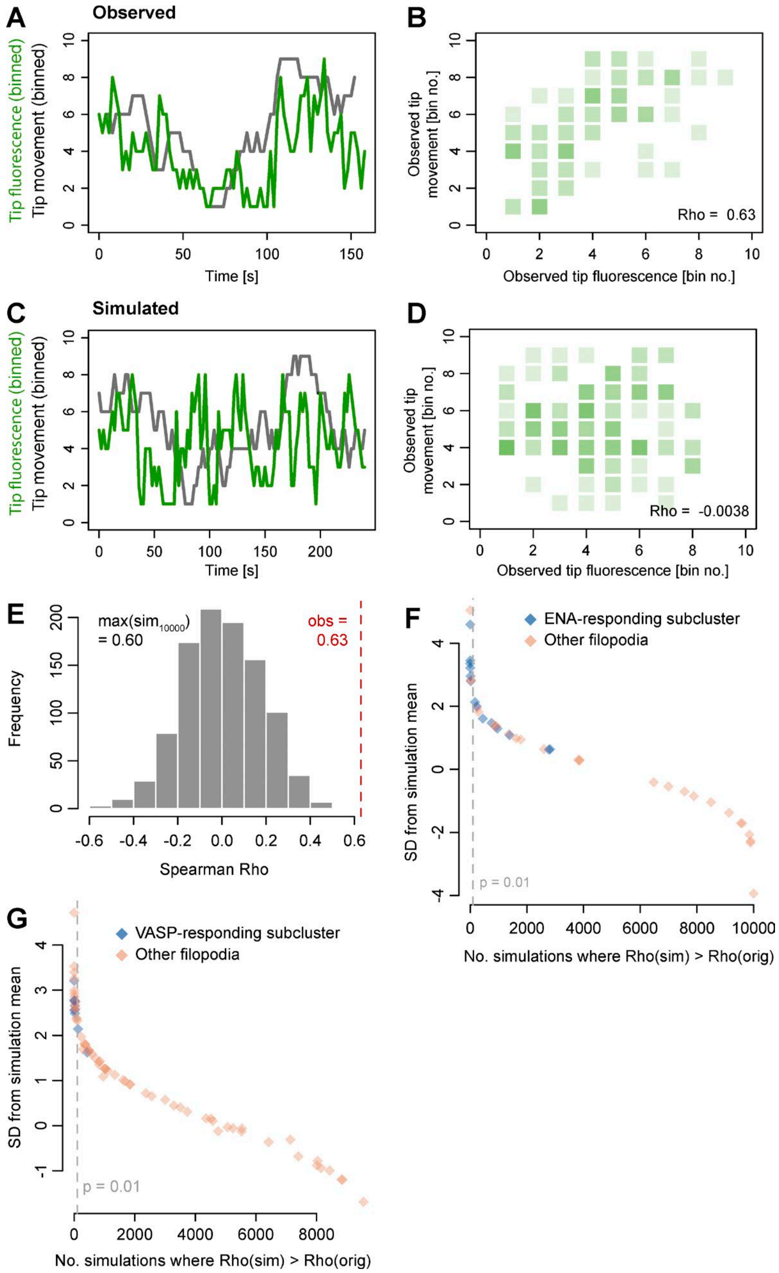

Figure S4. **A Monte Carlo Markov chain method confirms the low likelihood of observed correlations between ENA and VASP tip fluorescence and tip movement occurring by chance.** (A) Traces of tip fluorescence and tip movement of an example filopodium expressing mNeonGreen-ENA, binned into equal-sized intervals. (B) Binned fluorescence and movement measurements for the filopodium from A across its recorded lifetime showing a positive correlation between tip movement and tip fluorescence for this filopodium. (C) A simulated filopodium dataset based on Markov chain transition probabilities derived from the real filopodium data in A and B. (D) Correlation between simulated fluorescence and simulated movement across the time series for the simulation shown in C. (E) A histogram of correlation coefficients between simulated tip movement and simulated tip fluorescence for 10,000 simulations based on the transition probabilities of one filopodium shown in A and B (including the simulation shown in C and D). The mean of correlation coefficients of all simulations is zero; the maximum is 0.60, lower than the correlation coefficient of the measurements in the real (observed) dataset, 0.63 (corresponding to B). (F) A summary of results of 10,000 simulations per filopodium for all filopodia in the mNeonGreen-ENA–expressing dataset, providing the number of simulations whose correlation Rho (between simulated tip fluorescence and movement) exceeded the correlation Rho found in the original time series (x axis). y axis shows the distance of the observed correlation coefficient in original time series from the mean of correlation coefficients in all simulations for that filopodium, expressed as the number of SDs from the mean. Color code highlights the subcluster identity of each filopodium according to the clustering presented in Fig. 5 I. (G) As in F, for mNeonGreen-VASP–expressing filopodia. Color code highlights filopodium subcluster identity according to the clustering in Fig. 6 G.

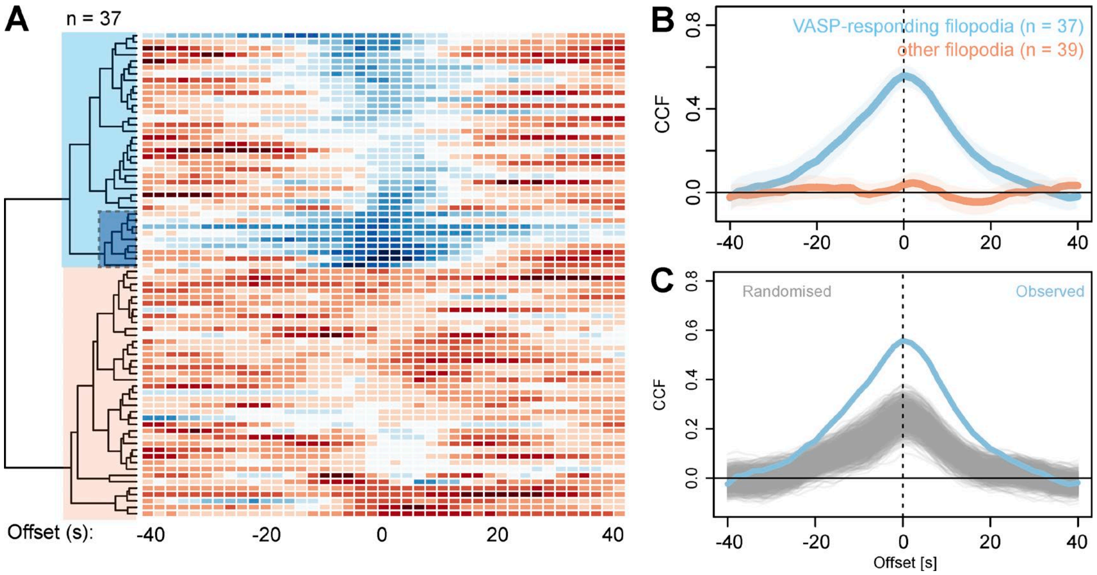

Figure S5. **Assessment of robustness in cross-correlation against subcluster size.** (A) Heatmap representing CCFs between mNeonGreen-VASP tip fluorescence and tip movement, directly duplicated from Fig. 6 G, analyzed here with a different subcluster selection threshold. In the new analysis, VASP-responding filopodia contain a much enlarged subset of filopodia (*n* = 37 out of 76; light blue block over dendrogram) compared with the analysis shown in Fig. 6 (G–J; *n* = 9 out of 76; shaded dark blue/dotted outline). (B) Mean and 95% confidence interval of the value of CCF computed collectively for VASP-responding filopodia (here, *n* = 37) and all other filopodia (*n* = 39) relative to offset. (C) Mean CCFs of the top correlating subcluster (with n size between 32 and 42) from each of 1000 randomizations of the VASP tip fluorescence and tip movement dataset. 0/1,000 randomizations recapitulate the observed mean top subcluster CCF (bootstrap $P < 0.001$).

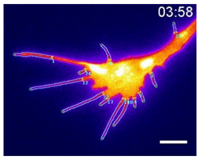

Video 1. **Time-lapse video of a *Xenopus* RGC growth cone, imaged with HILO illumination on a TIRF imaging system and segmented with Filopodyan.** Acquisition rate: 2 s per frame, playback speed: 12 frames per second (fps). False-color look-up table (LUT) "Fire": membrane marker GAP-RFP. Bar, 5 µm.

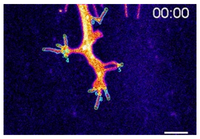

Video 2. ***Drosophila* tracheal cells imaged by line-scanning confocal microscopy, segmented with Filopodyan.** 15 s per frame; playback speed: 7 fps. False-color LUT Fire: membrane marker Cherry-CAAX. Bar, 5 µm.

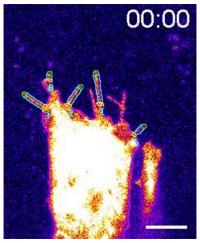

Video 3. ***Drosophila* leading edge cells during dorsal closure imaged by line-scanning confocal microscopy, segmented with Filopodyan.** Acquisition rate: 15 s per frame; playback speed: 7 fps. False-color LUT Fire: membrane marker cd8-mCherry. Bar, 5 µm.

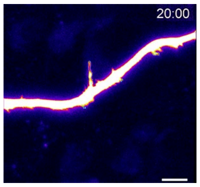

Video 4. **Dendritic filopodia in iPSC-derived human cortical neurons, imaged by two-photon microscopy and segmented with Filopodyan.** Acquisition rate: 2 min per frame; playback speed: 4 fps. False-color LUT Fire: cytoplasmic marker mNeonGreen. Bar, 5 µm.

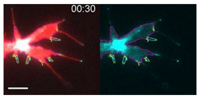

Video 5. **mNeonGreen-ENA localization during formation of new filopodia in an RGC growth cone imaged using HILO illumination.** Detected filopodia were filtered for newly forming filopodia (min start frame = 2). Acquisition rate: 2 s per frame; playback speed: 12 fps. Red, membrane marker GAP-RFP; cyan, mNeonGreen-ENA. Bar, 5 µm.

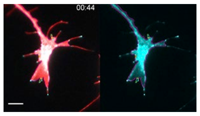

Video 6. **Localization of mNeonGreen-VASP before filopodia formation in an RGC growth cone imaged using HILO illumination (example from Fig. 4).** Detected filopodia were filtered to newly forming filopodia (min start frame = 2). Acquisition rate: 2 s per frame; playback speed: 12 fps. Red, membrane marker GAP-RFP; cyan, mNeonGreen-VASP. Bar, 5 µm.

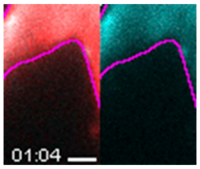

Video 7. **Localization of mNeonGreen-VASP in an RGC growth cone during formation of a single filopodium (inset from Video 6) in an RGC growth cone imaged with HILO illumination.** Acquisition rate: 2 s per frame; playback speed: 12 fps. Red, membrane marker GAP-RFP; cyan, mNeonGreen-VASP. Bar, 1 µm.

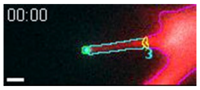

Video 8. **mNeonGreen-ENA localization in filopodia tips during extension (ENA-responding example from Fig. 5 C, rotated) in an RGC growth cone imaged with HILO illumination.** Acquisition rate: 2 s per frame; playback speed: 12 fps. Red, membrane marker GAP-RFP; cyan, mNeonGreen-ENA. Bar, 1 µm.

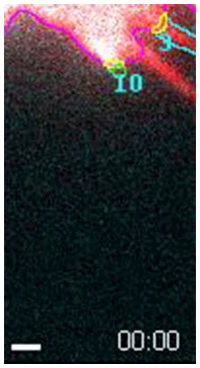

Video 9. **mNeonGreen-ENA localization in filopodia tips during extension (example from Fig. 5 F, rotated) in an RGC growth cone imaged with HILO illumination.** Acquisition rate: 2 s per frame; playback speed: 12 fps. Red, membrane marker GAP-RFP; cyan, mNeonGreen-ENA. Bar, 1 µm.

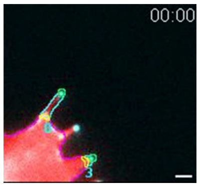

Video 10. **Two filopodia tips showing differential response to mNeonGreen-VASP accumulation (examples from Fig. 6, A and D) in an RGC growth cone imaged with HILO illumination.** Acquisition rate: 2 s per frame; playback speed: 12 fps. Red, membrane marker GAP-RFP; cyan, mNeonGreen-VASP. Bar, 1 µm.

Provided online is Table S1, which lists the full phenotypic measurements and associated statistics. The complete data tables outputted by Filopodyan of growth cone filopodia parameters and fluorescence intensities that were used as the source of further analysis are provided as a zipped dataset.
